# Importance of highly selective LC–MS/MS analysis for the accurate quantification of tamoxifen and its metabolites: focus on endoxifen and 4-hydroxytamoxifen

**DOI:** 10.1007/s10549-012-2000-1

**Published:** 2012-03-03

**Authors:** N. G. L. Jager, H. Rosing, S. C. Linn, J. H. M. Schellens, J. H. Beijnen

**Affiliations:** 1 Department of Pharmacy & Pharmacology, Slotervaart Hospital/The Netherlands Cancer Institute, Louwesweg 6, 1066 EC Amsterdam, The Netherlands; 2 Department of Medical Oncology, The Netherlands Cancer Institute, Plesmanlaan 121, 1066 CX Amsterdam, The Netherlands; 3 Department of Clinical Pharmacology, The Netherlands Cancer Institute, Plesmanlaan 121, 1066 CX Amsterdam, The Netherlands; 4 Department of Pharmaceutical Sciences, Faculty of Science, Utrecht University, 3508 TB Utrecht, The Netherlands

**Keywords:** Tamoxifen, Endoxifen, 4-Hydroxytamoxifen, Metabolite levels, LC–MS/MS analysis

## Abstract

The antiestrogenic effect of tamoxifen is mainly attributable to the active metabolites endoxifen and 4-hydroxytamoxifen. This effect is assumed to be concentration-dependent and therefore quantitative analysis of tamoxifen and metabolites for clinical studies and therapeutic drug monitoring is increasing. We investigated the large discrepancies in reported mean endoxifen and 4-hydroxytamoxifen concentrations. Two published LC–MS/MS methods are used to analyse a set of 75 serum samples from patients treated with tamoxifen. The method from Teunissen et al. (J Chrom B, 879:1677–1685, 2011) separates endoxifen and 4-hydroxytamoxifen from other tamoxifen metabolites with similar masses and fragmentation patterns. The second method, published by Gjerde et al. (J Chrom A, 1082:6–14, 2005) however lacks selectivity, resulting in a factor 2–3 overestimation of the endoxifen and 4-hydroxytamoxifen levels, respectively. We emphasize the use of highly selective LC–MS/MS methods for the quantification of tamoxifen and its metabolites in biological samples.

## Introduction

Tamoxifen is widely administered in the treatment and chemoprevention of estrogen receptor positive breast cancer, which accounts for about 60–70% of all breast cancers [1–3]. Tamoxifen is considered to be a prodrug that is converted into many metabolites. The most therapeutically active metabolites are *N*-desmethyl-4-hydroxytamoxifen (endoxifen) and 4-hydroxytamoxifen, being 30- to 100-fold more potent than tamoxifen itself. The antiestrogenic activities of endoxifen and 4-hydroxytamoxifen are similar, although endoxifen, unlike 4-hydroxytamoxifen, is also a potent inhibitor of aromatase and is present at a higher steady state concentration in patients than 4-hydroxytamoxifen [4–7].

The steady state levels of the active tamoxifen metabolites are proposed predictors of the clinical outcomes of tamoxifen treatment; it is suggested that there is a minimum concentration threshold above which endoxifen is effective against the recurrence of breast cancer. [8] It is well known from the literature that there is a considerable inter-patient variability in steady state levels of tamoxifen and its metabolites [5, 8–10]. However, the mean levels reported by recent studies [8, 10–14], that all included patients using 20 mg tamoxifen per day and analysed patient samples with liquid chromatography–tandem mass spectrometry (LC–MS/MS), differ more than expected purely based on the inter-patient variability. Three of these studies report mean endoxifen concentrations between 7.10 and 14.5 ng/mL and mean 4-hydroxytamoxifen levels between 1.55 and 2.25 ng/mL [9–11], similar to the levels we find in our laboratory, whereas another recent study reports concentrations twice as high [14]. Two studies from Norway, both using the LC–MS/MS assay developed by Gjerde et al. [15], report even higher concentrations; median concentrations of around 50 ng/mL for endoxifen and around 5.75 ng/mL for 4-hydroxytamoxifen [12, 13].

In this article we describe the investigation of these discrepancies, by analysing a set of 75 patient samples with the assay published by Gjerde et al. [15] and with an assay developed in our laboratory [16].

## Methods

### Patient samples

Serum samples were obtained in the period between December 2010 and September 2011 from patients treated with tamoxifen in the Netherlands Cancer Institute, Amsterdam, the Netherlands. The samples were collected in serum gel tubes and blood was allowed to coagulate for 30 min at room temperature. After coagulation, serum gel tubes were centrifuged for 10 min at 2,500–3,000 g (temperature was allowed to range from 4°C to ambient temperature). Serum was transferred into polypropylene tubes, which were stored at −70°C until the time of analysis.

### Extraction and measurement of tamoxifen and metabolites

Tamoxifen and its metabolites were analysed in 75 patient samples. All patient samples, 20 calibration standards and 6 quality control samples were handled according to the method described by Teunissen et al. [16]. A volume of 50 μL human serum was processed. Sample pre-treatment involved protein precipitation with acetonitrile. After mixing, samples were centrifuged and the clear supernatant was evaporated to dryness under a gentle stream of nitrogen (30°C). The extracts were reconstituted in acetonitrile—4 mM ammonium formate buffer pH 3.5 (3:7 v/v). The final extracts were analysed by two different LC–MS/MS assays, method 1 from Teunissen et al. [16] and method 2 from Gjerde et al. [15], during consecutive days.

### Method 1

The assay for the determination of tamoxifen (5–500 ng/mL), *N*-desmethyltamoxifen (10–1,000 ng/mL), (*E*)-endoxifen (1–100 ng/mL), (*Z*)-endoxifen (1–100 ng/mL), *N*-desmethyl-4′-hydroxytamoxifen (1–100 ng/mL), 4-hydroxytamoxifen (0.4–40 ng/mL) and 4′-hydroxytamoxifen (0.4–40 ng/mL), from Teunissen et al. [16] was used with slight modifications. A volume of 5 μL of the final extract was injected onto a Kinetex C18 100 Å column (100 × 4.6 mm ID) and detection was performed on a triple-quadrupole MS/MS detector with an electrospray ionization source (API4000, AB Sciex, Foster City, USA) operating in the positive ion mode. A partial validation was executed and all requirements for acceptance, as defined in the FDA and EMA guidelines on Bioanalytical Method Validation [17, 18] were fulfilled.

### Method 2

The assay for the determination of tamoxifen (5–500 ng/mL), *N*-desmethyltamoxifen (10–1000 ng/mL), endoxifen (1–100 ng/mL), and 4-hydroxytamoxifen (0.4–40 ng/mL) from Gjerde et al. [15] was used.

Online extraction was not executed in order to analyse the identical final extracts that were used when method 1 was applied. The flow rate was set at 0.8 mL/min, to obtain comparable retention times.

A volume of 5 μL of each sample was injected onto a Chromolith Performance RP 18-e column (100 × 4.6 mm ID) and a gradient elution similar to the separation mode of the method used by Gjerde et al. was applied. The separation was performed at room temperature and the autosampler was thermostatted at 7°C. Detection was performed on a triple-quadrupole MS/MS detector with an electrospray ionization source (API4000, AB Sciex, Foster City, USA) operating in the positive ion mode.

### Quantification of tamoxifen and metabolites

Tamoxifen, *N*-desmethyltamoxifen-HCl, *N*-desmethyl-4-hydroxytamoxifen (endoxifen, *E/Z* mixture 1:1), *N*-desmethyl-4′-hydroxytamoxifen, 4-hydroxytamoxifen, 4′-hydroxytamoxifen, tamoxifen-*N*-oxide, tamoxifen-d5, *N*-desmethyltamoxifen-d5, *N*-desmethyl-4-hydroxytamoxifen-d5 (endoxifen-d5, *E/Z* mixture 1:1) and 4-hydroxytamoxifen-d5 were purchased from Toronto Research Chemicals (North York, ON, Canada). The chemical structures of the analytes are shown in Table 1. Characterization of the peaks in patient samples was based on comparison with the retention times and MS fragmentation patterns of the reference standards. When no reference standard was available, identification was based on MS fragmentation and data found in the literature [10, 19]. The reference standard of endoxifen was a racemic mixture (1:1), resulting in baseline separated peaks when using method 1, but in a single peak when using method 2. For the quantification of (*Z*)-endoxifen in patient samples analysed with method 2, the analyte peak area of the calibration standards and quality control samples was divided by a factor 2.

**Table 1:** Trivial names, chemical structures and retention times of tamoxifen and metabolites with molecular mass 371.5, 357.5, 373.5 or 387.5 (reprinted and adjusted from Teunissen et al. [5], used with permission)


Trivial name	R_1_	R_2_	R_3_	R_4_	R_5_	R_6_	R_7_	Formula	Mol. Mass	Transition *(m/z)*	RT_1_ (min)	RT_2_ (min)
Tamoxifen	O–CH_2_–CH_2_–N(CH_3_)_2_	CH_2_–CH_3_	H	H	H	H	H	C_26_H_29_NO	371.5	372/72	8.00	2.99
*N*-desmethyltamoxifen	O–CH_2_–CH_2_–NH–CH_3_	CH_2_–CH_3_	H	H	H	H	H	C_25_H_27_NO	357.5	358/58	7.91	2.96
*N*-desmethyl-α-hydroxytamoxifen^a^	O–CH_2_–CH_2_–NH–CH_3_	CH(OH)–CH_3_	H	H	H	H	H	C_25_H_27_NO_2_	373.5	374/58	6.65	2.81
*N*-desmethyl-4-hydroxytamoxifen (Endoxifen)	O–CH_2_–CH_2_–NH–CH_3_	CH_2_–CH_3_	H	H	OH	H	H	C_25_H_27_NO_2_	373.5	374/58	5.79	2.81
*N*-desmethyl-3-hydroxytamoxifen^a^	O–CH_2_–CH_2_–NH–CH_3_	CH_2_–CH_3_	H	OH	H	H	H	C_25_H_27_NO_2_	373.5	374/58	5.85	2.81
*N*-desmethyl-4′-hydroxytamoxifen	O–CH_2_–CH_2_–NH–CH_3_	CH_2_–CH_3_	H	H	H	H	OH	C_25_H_27_NO_2_	373.5	374/58	6.41	2.81
α-Hydroxytamoxifen^a^	O–CH_2_–CH_2_–N(CH_3_)_2_	CH(OH)–CH_3_	H	H	H	H	H	C_26_H_29_NO_2_	387.5	388/72	3.91	2.84
4-Hydroxytamoxifen	O–CH_2_–CH_2_–N(CH_3_)_2_	CH_2_–CH_3_	H	H	OH	H	H	C_26_H_29_NO_2_	387.5	388/72	6.03	2.84
3-Hydroxytamoxifen^a^	O–CH_2_–CH_2_–N(CH_3_)_2_	CH_2_–CH_3_	H	OH	H	H	H	C_26_H_29_NO_2_	387.5	388/72	6.17	2.84
4′-Hydroxytamoxifen	O–CH_2_–CH_2_–N(CH_3_)_2_	CH_2_–CH_3_	H	H	H	H	OH	C_26_H_29_NO_2_	387.5	388/72	6.66	2.84
Tamoxifen-*N*-oxide	O–CH_2_–CH_2_–NO(CH_3_)_2_	CH_2_–CH_3_	H	H	H	H	H	C_26_H_29_NO_2_	387.5	388/72	8.32	3.06
β-Hydroxytamoxifen^b^	O–CH_2_–CH_2_–N(CH_3_)_2_	CH_2_–CH_2_–OH	H	H	H	H	H	C_26_H_29_NO_2_	387.5	388/72	–	–
2-Hydroxytamoxifen^b^	O–CH_2_–CH_2_–N(CH_3_)_2_	CH_2_–CH_3_	OH	H	H	H	H	C_26_H_29_NO_2_	387.5	388/72	–	–
1,2-Epoxytamoxifen^b^	O–CH_2_–CH_2_–N(CH_3_)_2_	CH_2_–CH_3_	H	H	H	H	H	C_26_H_29_NO_2_	387.5	388/72	–	–

*RT*_1_ retention time obtained with method 1, based on the method developed by Teunissen et al. [16]. *RT*_2_ retention time obtained with method 2, based on the method developed by Gjerde et al. [15]

^a^No reference standard available, identification was based on MS fragmentation and data found in the literature [10, 19]

^b^The levels of these metabolites are below the LLOD of current LC–MS platforms [11, 20], therefore retention times are unknown

Multiple reaction monitoring chromatograms were acquired at unit resolution (0.7 Da) for quantification.

## Results and discussion

There are large differences in reported mean steady-state concentrations of the therapeutically active tamoxifen metabolites, endoxifen and 4-hydroxytamoxifen. These discrepancies can only partly be assigned to inter-patient variability in the biotransformation of tamoxifen. For this article, we investigated the bioanalytical variability, by analysing a set of 75 patient samples with two different LC–MS/MS methods; method 1 from Teunissen et al. [16] and method 2 from Gjerde et al. [15]. The bioanalytical data were accepted for both methods, since the back-calculated concentrations of the calibration standards and quality control samples were all within ±15%. The results are presented in Table 2.

**Table 2:** Mean concentrations of tamoxifen and three of its metabolites analysed with the two described methods, from serum samples of 75 patients treated with tamoxifen

	Mean concentration (ng/mL)	
Analyte	Method 1 [16] ± s.d.	Method 2 [15] ± s.d.
Tamoxifen	99.7 ± 39.3	103.3 ± 40.4
*N*-desmethyltamoxifen	184.0 ± 74.7	187.1 ± 77.9
Endoxifen	9.0 ± 4.5	18.1 ± 6.4
4-Hydroxytamoxifen	1.7 ± 0.7	4.6 ± 1.7

### Tamoxifen and *N*-desmethyltamoxifen

The measured concentration of tamoxifen and *N*-desmethyltamoxifen in each serum sample was very similar for both methods (Fig. 2), resulting in comparable mean concentrations (Table 2). There are no tamoxifen metabolites described in the literature [6, 20] that have molecular masses similar to tamoxifen or *N*-desmethyltamoxifen, therefore co-elution of tamoxifen analogues with fragmentation patterns similar to tamoxifen or *N*-desmethyltamoxifen is not expected.

### Endoxifen (*m*/*z* 374 → 58)

There are several metabolites with close resemblance in molecular structure to endoxifen (Table 1). These compounds also have similar molecular masses and fragmentation patterns, making chromatographic separation of crucial importance for selective analysis.

**Fig. 1:**
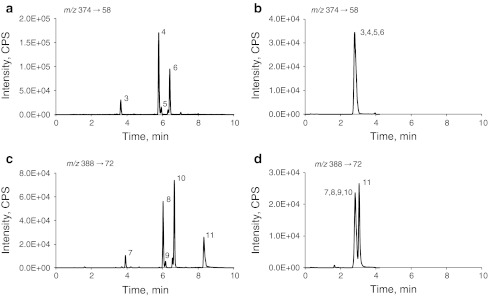
Representative LC–MS/MS chromatograms obtained from a study patient sample. Chromatograms **a** and **b** were obtained with method 1, from Teunissen et al. [16], and method 2, from Gjerde et al. [15], respectively, when *m*/*z* 374 → 58 was monitored. Chromatograms **c** and **d** were obtained with method 1 and 2, respectively, when *m*/*z* 388 → 72 was monitored Peak numbers correspond with metabolite numbers in Table 1

**Fig. 2:**
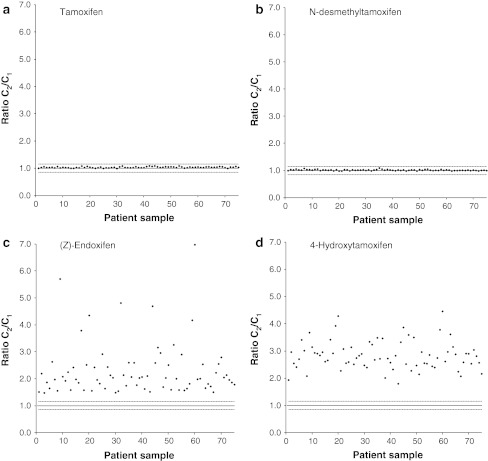
Ratio of the measured concentrations obtained with method 1, C_1_, and method 2, C_2_, in 75 patient samples for tamoxifen (**a**), *N*-desmethyltamoxifen (**b**), *(Z)*-endoxifen (**c**) and 4-hydroxytamoxifen (**d**). The solid line represents a ratio of 1.0 (i.e. equal measured concentrations) and the dotted lines represent the (bioanalytically accepted) ±15% deviation from method 2 in comparison with method 1

The chromatogram obtained with the method from Teunissen et al. [16] (Fig. 1a) shows separate peaks for the metabolites with mass transition 374/58, whereas the chromatogram obtained with the method from Gjerde et al. [15] (Fig. 1b) shows only a single peak, consisting of *N*-desmethyl-α-hydroxytamoxifen, endoxifen, *N*-desmethyl-3-hydroxytamoxifen and *N*-desmethyl-4′-hydroxytamoxifen eluting at the same retention time. This lack of selectivity leads to a consequent overestimation of the endoxifen level of around a factor 2 (Fig. 2; Table 2). Furthermore, method 1 separates (*E*)-endoxifen from the therapeutically active (*Z*)-endoxifen, whereas method 2 does not separate these isoforms. However, for all 75 patient samples the (*E*)-endoxifen level was below the lower limit of quantitation (1.0 ng/mL), which is in agreement with the literature [10, 19].

### 4-Hydroxytamoxifen (*m*/*z* 388 → 72)

As shown in Table 1, there are at least seven tamoxifen metabolites with masses and fragmentation patterns similar to 4-hydroxytamoxifen. From these metabolites, the levels of β-hydroxytamoxifen, 2-hydroxytamoxifen and 1,2-epoxytamoxifen are below the lower limit of detection (LLOD) of current LC–MS platforms (±0.05 ng/mL) [11, 20]. The chromatogram obtained with method 1 (Fig. 1c) shows separate peaks for the other four metabolites with mass transition 388/72, whereas the chromatogram obtained with method 2 (Fig. 1d) shows only two separated peaks. Tamoxifen-*N*-oxide elutes at 3.06 min and α-hydroxytamoxifen, 4-hydroxytamoxifen, 3-hydroxytamoxifen and 4′-hydroxytamoxifen are co-eluting at 2.84 min. This co-elution leads to a consequent overestimation of the 4-hydroxytamoxifen levels of around a factor 3 (Fig. 2) and therefore the mean 4-hydroxytamoxifen concentration obtained with method 2 is a factor 3 higher (Table 2).

The results obtained with method 1 are in good agreement with the levels reported by three recent published studies. The analytical methods used in these studies all separated endoxifen and 4-hydroxytamoxifen from compounds with similar masses and fragmentation patterns [8, 10, 11].

When investigating correlations between the levels of the active tamoxifen metabolites and efficacy and toxicity parameters, it is crucial to distinguish between the active metabolites and the 4′-hydroxylated metabolites, which are about ten times less active than 4-hydroxytamoxifen and endoxifen. [10, 11] Also, for therapeutic drug monitoring based on reaching a sufficient endoxifen level, it is important to use a highly selective analysis in order to accurately quantify endoxifen in the patient sample.

## Conclusions

This article demonstrates that high selectivity is of major importance for the analysis of tamoxifen metabolites, some of which show marked resemblance in molecular structure and have similar masses and fragmentation patterns. Lack of high selectivity results in an overestimation of the concentration of the therapeutically active metabolites, endoxifen and 4-hydroxytamoxifen, in patient samples.
